# Discrimination of the SARS–CoV-2 strains using of coloured s-LASCA-imaging of GB-speckles, developed for the gene “S” nucleotide sequences

**DOI:** 10.12688/f1000research.53214.3

**Published:** 2021-11-08

**Authors:** Onega Ulianova, Yury Saltykov, Sergey Ulyanov, Sergey Zaytsev, Alexander Ulyanov, Valentina Feodorova

**Affiliations:** 1 Department of Medical Physics, Saratov State University, Saratov, 410012, Russian Federation; 2 Laboratory for Molecular Biology and NanoBioTechnology, Federal Research Center for Virology and Microbiology, Branch in Saratov, Saratov, 410076, Russian Federation

**Keywords:** LASCA, SARS–CoV-2, GB-speckles, gene

## Abstract

**Background**: A recent bioinformatics technique involves changing nucleotide sequences into 2D speckles. This technique produces speckles called GB-speckles (Gene Based speckles). All classical strategies of speckle-optics, namely speckle-interferometry, subtraction of speckle-images as well as speckle-correlometry have been inferred for processing of GB-speckles. This indicates the considerable improvement in the present tools of bioinformatics.

**Methods**: Colour s-LASCA imaging of virtual laser GB-speckles, a new method of high discrimination and typing of pathogenic viruses, has been developed. This method has been adapted to the detecting of natural mutations in nucleotide sequences, related to the spike glycoprotein (coding the gene «S») of SARS–CoV-2 gene as the molecular target.

**Results**: The rate of the colouring images of virtual laser GB-speckles generated by s-LASCA can be described by the specific value of R. If the nucleotide sequences compared utilizing this approach the relevant images are completely identical, then the three components of the resulting colour image will be identical, and therefore the value of R will be equal to zero. However, if there are at least minimal differences in the matched nucleotide sequences, then the value of R will be positive.

**Conclusion**: The high effectiveness of an application of the colour images of GB-speckles that were generated by s-LASCA- has been demonstrated for discrimination between different variants of the SARS–CoV-2 spike glycoprotein gene.

## Introduction

As it is well known, if laser light diffracts on random objects, then laser speckles are formed. ^1- 3^ Recently, the possibility of transforming a nucleotide sequence into a pattern of 2D speckles had been demonstrated. ^4- 9^ This new type of speckle pattern has been called “GB-speckles” (gene-based speckles). ^5, 7, 9^ Changes within in the structure of the GB-speckles can reflect even negligible changes in the nucleotide sequence, caused by inartificial mutations. This allows detection of single-nucleotide polymorphisms (SNP) using virtual GB-speckles with outstanding precision. In addition, it offers unlimited potential of improving the diagnosis’ accuracy by increasing the Fourier transform area. ^10^

Essential advancement in the area of GB-speckles has been reported in previous years. According to previously published reports, ^4- 9, 11^ implementation of speckle-optics methods, like speckle-interferometry and subtraction of speckle-images as well as speckle-correlometry for processing of GB-speckles, provides considerable progress in the current bioinformatics toolbox. This can become crucial to significantly improve existing routine methods of laboratory diagnostics of infectious diseases. GB-speckles as a technique opens the door to the new horizons in digital biology. ^12, 13^

Recently, model GB-speckle patterns of nucleotide sequences of the *omp1* genes for two different of *Chlamydia* spp., such as *Chlamydia trachomatis* and *Chlamydia psittaci* of at least six genovars (D, E, F, G, J and K) have been composed. ^4, 5^ Probability density functions and correlation properties of spatial intensity fluctuations for the relevant GB-speckle patterns have been studied. ^5- 7^ As it has been shown in previous studies, ^4- 7, 9^ the presence of inartificial mutations in analysed strains, including single SNP cases, can be easily defined using methods of speckle-optics. ^4- 7, 9^ More recently, the encoding algorithm’s optimization for nucleotide sequences of *C. trachomatis* into two-dimensional GB-speckle pattern had been carried out; ^4, 6^ and speckle-interferometric technique may give rise the ultra-fast optical processors of DNA sequences. ^4^ This is ensured by the development of the exclusive system of interferential fringes which are generated by the model interference pattern led by the existence of any type of mutations. Additionally, the method of virtual phase-shifting speckle-interferometry was reported to be efficacious ^11^ to investigate of polymorphism of the *C. trachomatis omp1* gene. This approach allowed the detection of the *C. trachomatis omp1* gene with SNPs, including both a single SNP and a combination of several SNPs in the bacterial strains with genetic mutations (11 known subtypes in total) had been developed. ^6^

The format of GB-speckles had been successfully applied to transform the nucleotide sequences of the genes expressing the serine proteases, the well-known Omptin family proteins within the *Enterobacteriaceae.* These proteins have been found on the surface of several bacterial agents causing different enteric infections, such as salmonellosis, shigelosis, yersiniosis, and escherichiosis. ^7^ Further, the phase and the relevant two-dimensional distributions of the intensity of GB-speckles in various strains of viral pathogens, namely of lumpy skin disease virus of cattle, LSDV, and also for sheep-pox virus, SPPV have been obtained. ^8^ Additionally, interference patterns for generated the specific superposition in the relevant fields of GB-speckle and the certain difference in their images have been successfully investigated to reveal a minimal discrimination between the initial viral nucleotide sequences.

A new bioinformatics approach has been proposed very recently: ^14^ GB-speckles processing *via* an *s-LASCA* technique (from the spatial Laser Speckle Contrast Analysis) application. As it had been demonstrated, it is possible to extend affectability of the proposed approach comparing to current bioinformatics strategies ^15^ using *s-LASCA* imaging in the GB-speckles’ processing. It had been shown in Ref. 16, that the GB-speckles’ generation combined with *s-LASCA* imaging method are very effective to analyze nucleotide polymorphism in several genes of *C. trachomatis.*

This paper is devoted to development of advantageously new technique: the coloured *s-LASCA* imaging of GB-speckles. Such a technique is an improved version of previously suggested “greyscale” *s-LASCA* imaging that was recently developed especially for GB-speckles. Nucleotide sequences for some target genes SARS–CoV-2 have been successfully processed using coloured *s-LASCA*-imaging. Natural mutations in the comparing genes have been reliably and accurately detected.

## Methods

### Nucleotide sequences under consideration.

Seven nucleotide sequences of spike glycoprotein of SARS-CoV-2, namely:

the gene#1. hCoV-19/cat/USA/TX-TAMU-078/2020 (Accession ID: EPI ISL 699509),

the gene#2. hCoV-19/cat/Russia/RII-LEN-22246S/2021 (Accession ID: EPI ISL 811147),

the gene#3. hCoV-19/cat/Greece/2K/2020 (Accession ID: EPI ISL 717979),

the gene#4. hCoV-19/Wuhan/WIV04/2019 (Accession ID: EPI ISL 402124),

the gene#5. hCoV-19/England/QEUH-B11766/2020 (Accession ID: EPI ISL 642476),

the gene#6. hCoV19/South Africa/KRISP-EC-K005299/2020 (Accession ID: EPI ISL 678597),

the gene#7. hCoV-19/Russia/MOS-CRIE-13604226/2020 (Accession ID: EPI ISL 754198).

have been compared on the base of analysis of GB-speckles. The official reference sequences were taken from the GISAID database.

Algorithm for the total conversion of a nucleotide sequence to a colour GB speckle structure, processed by *s-LASCA* imaging technique

### Initial processing of nucleotide sequence

First, the sequence of the letters derived from the original one-dimensional nucleotide sequence was converted into the sequence of numbers in accordance with the following rule: ^4^

A→1; C→2; G→3; T→4. (1)

It is critical to emphasize that the specific relationship between the letters and numbers in this case is not critical as used earlier; ^6^ thus, other rules could have been applied to the encoding, for instance:

C→1; G→2; T→3; A→4. (2)

or

T→1; A→2; C→3; G→4. (3)

Next, all possible triad combination are generated. As a result, a complete set of all triads is formed:

(1 1 1), (1 1 2), (1 1 3), (1 1 4), (1 2 1), (1 2 2), (1 2 3), (1 2 4), (1 3 1), ... , (4 4 4). (4)

The number of all possible combinations of four numbers combined in triads is 64.

Then, a discrete magnitude, h, is allotted to each triad in accordance with the simple algorithm described previously. ^4^ This algorithm was implemented in Matlab R2015a ( **RRID:SCR_001622**); an open access alternative is Julia. The value of h is a positive integer, varying in the range from 1 to 64. In this case, each triad from the original nucleotide sequence is associated with only one h value. So, for example, the combination (1 1 1) conforms to the value h = 1, (1 1 2) corresponds to h = 2, (1 1 3) conforms to h = 3, (1 1 4) conforms to h = 4, (1 2 1) conforms to h = 5, (1 2 2) conforms to h = 6, and so on. Finally, the latest combination (4 4 4) conforms to the value h = 64. Finally, a square matrix H _n,m_ was formed by a one-dimensional array h. The physical significance of the shaped matrix H _n,m_ is that each of its elements represents the local height of some virtual rough surface corresponding to the local content of the analyzed genetic construction. The resulting virtual rough surfaces could be used to model original speckle structures corresponding to diverse particular nucleotide sequences.

The two-dimensional speckle patterns that corresponded to each specific sequence was generated with the use the diffraction of a coherent beam with a square cross-section profile on a virtual scattering surface with a microrelief described by the matrix H _n,m_. At each point of the virtual diffuser (in the beam scattering plane), some phase modulation U _n,m_ = exp(−2π *j* H _n,m_/64) is introduced ( *j* is an imaginary unit). The surface is illuminated at the normal incidence of the beam; the phase in the illuminating beam was a constant value.

It is assumed that speckles are formed in the far diffraction zone and described in the Fraunhofer approximation. In this case, the expression for the amplitude of the scattered field is the Fourier transform of the field in the diffraction plane, evaluated at frequencies spaces ^10^

$$F_{x}=X_{o}/(z*\lambda ),\;\;\;\;F_{y}=Y_{o}/(z*\lambda ),$$ (5)

where *X*_*o*_ and *Y*_*o*_ are the coordinates in the observation plane, *z* is the distance between the scattering plane and the observation plane, *λ* is the wavelength. The illuminating radiation is completely monochromatic, thus, *λ* = const. In this situation, the structure of speckles does not depend on the wavelength and *z.* Only the sizes of GB-speckles depend on these values, the average size of which is determined by the ratio:

d∼3*z*λ/a (6)

where *a* is the size of the illuminated fragment of virtual surface. ^17^ It is important to emphasize that the ratio *λ*/ *a* characterizes the diffraction angular divergence of a laser beam in the far field, and the product of this divergence angle by the light traveled distance *z* is equal to the lateral size of the beam. Thus, it can be seen that the diameter of the undisturbed laser beam (namely, this value is on the right side of the expression (6)) and the average speckle size are approximately equal to each other in any observation plane. In other words, when the parameters *z* and *λ* change, a proportional change in the size of all speckles occurs synchronously. At the same time, the structure of speckle-patterns in all observation planes are completely similar, only their scale changes from plane to plane, but not the shape of the speckles or their location in the speckle pattern.

### Generating GB-speckles

The procedure for transcoding the original nucleotide sequence into a GB-speckle structure using the example of the hCoV-19/cat/USA/TX-TAMU-078/2020|2020-07-29 gene (the gene #1) is shown below.

The original nucleotide sequence is as follows:

ATGTTTGTTTTTCTTGTTTTATTGCCACTAGTCTCTAGTCAGTGTGTTAATCTTACAACCAGAACTCAATTACCCCCTGCATACACTAATTCTTTCACACGTGGTGTTTATTACCCTGACAAAGTTTTCAGATCCTCAGTTTTACATTCAACTCAGGACTTGTTCTTACCTTTCTTTTCCAATGTTACTTGGTTCCATGCTATACATGTCTCTGGGACCAATGGTACTAAGAGGTTTGATAACCCTGTCCTACCATTTAATGATGGTGTTTATTTTGCTTCCACTGAGAAGTCTAACATAATAAGAGGCTGGATTTTTGGTACTACTTTAGATTCGAAGACCCAGTCCCTACTTATTGTTAATAACGCTACTAATGTTGTTATTAAAGTCTGTGAATTTCAATTTTGTAATGATCCATTTTTGGGTGTTTATTACCACAAAAACAACAAAAGTTGGATGGAAAGTGAGTTCAGAGTTTATTCTAGTGCGAATAATTGCACTTTTGAATATGTCTCTCAGCCTTTTCTTATGGACCTTGAAGGAAAACAGGGTAATTTCAAAAATCTTAGGGAATTTGTGTTTAAGAATATTGATGGTTATTTTAAAATATATTCTAAGCACACGCCTATTAATTTAGTGCGTGATCTCCCTCAGGGTTTTTCGGCTTTAGAACCATTGGTAGATTTGCCAATAGGTATTAACATCACTAGGTTTCAAACTTTACTTGCTTTACATAGAAGTTATTTGACTCCTGGTGATTCTTCTTCAGGTTGGACAGCTGGTGCTGCAGCTTATTATGTGGGTTATCTTCAACCTAGGACTTTTCTATTAAAATATAATGAAAATGGAACCATTACAGATGCTGTAGACTGTGCACTTGACCCTCTCTCAGAAGCAAAGTGTACGTTGAAATCCTTCACTGTAGAAAAAGGAATCTATCAAACTTCTAACTTTAGAGTCCAACCAACAGAATCTATTGTTAGATTTCCTAATATTACAAACTTGTGCCCTTTTGGTGAAGTTTTTAACGCCACCAGATTTGCATCTGTTTATGCTTGGAACAGGAAGAGAATCAGCAACTGTGTTGCTGATTATTCTGTCCTATATAATTCCGCATCATTTTCCACTTTTAAGTGTTATGGAGTGTCTCCTACTAAATTAAATGATCTCTGCTTTACTAATGTCTATGCAGATTCATTTGTAATTAGAGGTGATGAAGTCAGACAAATCGCTCCAGGGCAAACTGGAAAGATTGCTGATTATAATTATAAATTACCAGATGATTTTACAGGCTGCGTTATAGCTTGGAATTCTAACAATCTTGATTCTAAGGTTGGTGGTAATTATAATTACCTGTATAGATTGTTTAGGAAGTCTAATCTCAAACCTTTTGAGAGAGATATTTCAACTGAAATCTATCAGGCCGGTAGCACACCTTGTAATGGTGTTGAAGGTTTTAATTGTTACTTTCCTTTACAATCATATGGTTTCCAACCCACTAATGGTGTTGGTTACCAACCATACAGAGTAGTAGTACTTTCTTTTGAACTTCTACATGCACCAGCAACTGTTTGTGGACCTAAAAAGTCTACTAATTTGGTTAAAAACAAATGTGTCAATTTCAACTTCAATGGTTTAACAGGCACAGGTGTTCTTACTGAGTCTAACAAAAAGTTTCTGCCTTTCCAACAATTTGGCAGAGACATTGCTGACACTACTGATGCTGTCCGTGATCCACAGACACTTGAGATTCTTGACATTACACCATGTTCTTTTGGTGGTGTCAGTGTTATAACACCAGGAACAAATACTTCTAACCAGGTTGCTGTTCTTTATCAGGGTGTTAACTGCACAGAAGTCCCTGTTGCTATTCATGCAGATCAACTTACTCCTACTTGGCGTGTTTATTCTACAGGTTCTAATGTTTTTCAAACACGTGCAGGCTGTTTAATAGGGGCTGAACATGTCAACAACTCATATGAGTGTGACATACCCATTGGTGCAGGTATATGCGCTAGTTATCAGACTCAGACTAATTCTCCTCGGCGGGCACGTAGTGTAGCTAGTCAATCCATCATTGCCTACACTATGTCACTTGGTGCAGAAAATTCAGTTGCTTACTCTAATAACTCTATTGCCATACCCACAAATTTTACTATTAGTGTTACCACAGAAATTCTACCAGTGTCTATGACCAAGACATCAGTAGATTGTACAATGTACATTTGTGGTGATTCAACTGAATGCAGCAATCTTTTGTTGCAATATGGCAGTTTTTGTACACAATTAAACCGTGCTTTAACTGGAATAGCTGTTGAACAAGACAAAAACACCCAAGAAGTTTTTGCACAAGTCAAACAAATTTACAAAACACCACCAATTAAAGATTTTGGTGGTTTTAATTTTTCACAAATATTACCAGATCCATCAAAACCAAGCAAGAGGTCATTTATTGAAGATCTACTTTTCAACAAAGTGACACTTGCAGATGCTGGCTTCATCAAACAATATGGTGATTGCCTTGGTGATATTGCTGCTAGAGACCTCATTTGTGCACAAAAGTTTAACGGCCTTACTGTTTTGCCACCTTTGCTCACAGATGAAATGATTGCTCAATACACTTCTGCACTGTTAGCGGGTACAATCACTTCTGGTTGGACCTTTGGTGCAGGTGCTGCATTACAAATACCATTTGCTATGCAAATGGCTTATAGGTTTAATGGTATTGGAGTTACACAGAATGTTCTCTATGAGAACCAAAAATTGATTGCCAACCAATTTAATAGTGCTATTGGCAAAATTCAAGACTCACTTTCTTCCACAGCAAGTGCACTTGGAAAACTTCAAGATGTGGTCAACCAAAATGCACAAGCTTTAAACACGCTTGTTAAACAACTTAGCTCCAATTTTGGTGCAATTTCAAGTGTTTTAAATGATATCCTTTCACGTCTTGACAAAGTTGAGGCTGAAGTGCAAATTGATAGGTTGATCACAGGCAGACTTCAAAGTTTGCAGACATATGTGACTCAACAATTAATTAGAGCTGCAGAAATCAGAGCTTCTGCTAATCTTGCTGCTACTAAAATGTCAGAGTGTGTACTTGGACAATCAAAAAGAGTTGATTTTTGTGGAAAGGGCTATCATCTTATGTCCTTCCCTCAGTCAGCACCTCATGGTGTAGTCTTCTTGCATGTGACTTATGTCCCTGCACAAGAAAAGAACTTCACAACTGCTCCTGCCATTTGTCATGATGGAAAAGCACACTTTCCTCGTGAAGGTGTCTTTGTTTCAAATGGCACACACTGGTTTGTAACACAAAGGAATTTTTATGAACCACAAATCATTACTACAGACAACACATTTGTGTCTGGTAACTGTGATGTTGTAATAGGAATTGTCAACAACACAGTTTATGATCCTTTGCAACCTGAATTAGACTCATTCAAGGAGGAGTTAGATAAATATTTTAAGAATCATACATCACCAGATGTTGATTTAGGTGACATCTCTGGCATTAATGCTTCAGTTGTAAACATTCAAAAAGAAATTGACCGCCTCAATGAGGTTGCCAAGAATTTAAATGAATCTCTCATCGATCTCCAAGAACTTGGAAAGTATGAGCAGTATATAAAATGGCCATGGTACATTTGGCTAGGTTTTATAGCTGGCTTGATTGCCATAGTAATGGTGACAATTATGCTTTGCTGTATGACCAGTTGCTGTAGTTGTCTCAAGGGCTGTTGTTCTTGTGGATCCTGCTGCAAATTTGATGAAGACGACTCTGAGCCAGTGCTCAAAGGAGTCAAATTACATTACACATAA (7)

After converting a sequence of letters into a sequence of numbers in accordance with the algorithm described by rule (1) described previously, the nucleotide sequence takes the following form:

143444344444244344441443221241342424134213434344114244121122131124211441222224321412124114424442121234334344414412224312111344442131422421344441214421124213312443442441224442444422114344124433442214324141214342424333122114334124113133444314112224342241221444114314334344414444324422124313113424112141141131332433144444334124124441314423113122213422241244144344114112324124114344344144111342434311444211444434114314221444443334344414412212111112112111134433143311134313442131344414424134323114114432124444311414342424213224444244143312244311331111213334114442111114244133311444343444113114144314334414444111141414424113212123224144114441343234314242224213334444423324441311221443341314443221141334144112142124133444211124441244324441214131134414443124224334314424424421334433121324334324321324414414343334414244211224133124444241441111414114311114331122144121314324341312434321244312224242421311321113434123443111422442124341311111331142414211124424112444131342211221121311424144344131444224114144121112443432224444334311344444112322122131444321424344414324433112133113131142132112434344324314414424342241414114422321421444422124444113434414331343424224124111441114314242432444124114342414321314421444341144131334314311342131211142324221333211124331113144324314414114414111441221314314444121332432344141324433114424112114244314424113344334334114414114412243414131443444133113424114242111224444313131314144421124311142414213322334132121224434114334344311334444114434412444224441211421414334442211222124114334344334412211221412131341341341244424444311244241214321221321124344434331224111113424124114443344111112111434342114442112442114334441121332121334344244124313424112111113444243224442211211444332131312144324312124124314324342234314221213121244313144244312144121221434424444334334342134344141121221331121114124424112213344324344244414213334344112432121311342224344324144214321314211244124224124433234344414424121334424114344444211121234321332434441141333324311214342112112421414313434312141222144334321334141432324134414213124213124114424224233233321234134341324134211422142144322412124143421244334321311114421344324412424114112424144322141222121114444124144134344122121311144241221343424143122113121421341314434121143412144434334314421124311432132114244443443211414332134444434121211441112234324441124331141324344311211312111112122211311344444321211342111211144412111121221221144111314444334334444114444421211141441221314221421111221132113133421444144311314241244442112111343121244321314324332442142111211414334314432244334314144324324131312242144434321211113444112332244124344443221224443242121314311143144324211412124424321243441323334121142124424334433122444334321334324321441211141221444324143211143324414133444114334144331344121213114344242414313112211111443144322112211444114134324144332111144211312421244424422121321134321244331111244211314343342112211114321211324441112123244344111211244132422114444334321144421134344441114314142244421234244312111344313324311343211144314133443142121332131244211134443213121414343124211211441144131324321311142131324424324114244324324124111143421313434341244331211421111131344314444434331113332414214244143422442224213421321224214334341342442443214343124414342224321211311113112442121124324224322144434214314331111321212444224234311334342444344421114332121212433444341121211133114444414311221211142144124121312112121444343424334112434314344341141331144342112112121344414314224443211224311441312421442113313313441314111414444113114214121421221314344314441334312142424332144114324421344341112144211111311144312232242114313344322113114441114311424242142314242211311244331113414313213414141111433221433412144433241334444141324332443144322141341143343121144143244432434143122134432434134434242113332434434424434331422432432111444314311312312424313221343242111331342111441214412121411 (8)

**Figure 1.**
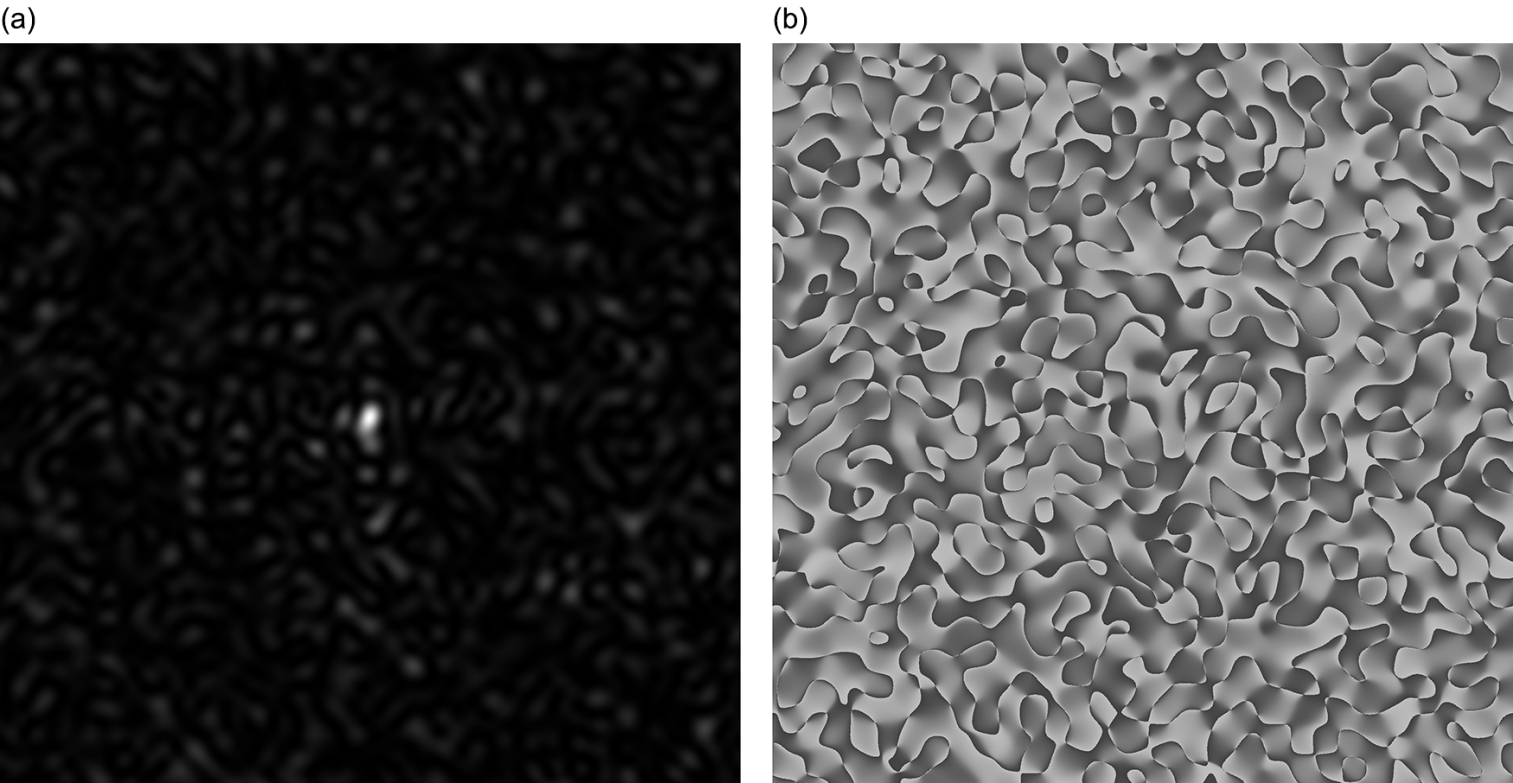
GB-speckles: (a) Speckle pattern for 2D intensity distribution, (b) Speckle pattern for 2D phase distribution.

As a result of diffraction of coherent beam on the phase screen (the virtual heights of the irregularities presented in the table (6)) with a square cross-section is formed GB-speckle-structure of two-dimensional intensity distribution, see Figure 1a. Two-dimensional phase distribution GB, the speckle structure is shown in Figure 1b.

Important to emphasize, that experimental studies were not carried out in this work, only computer modeling. The scheme for calculating GB-speckles during radiation diffraction on a virtual scattering surface is described in detail in the work. ^9^

### *s-LASCA* imaging of GB-speckles

*s-LASCA* strategy has been connected for handling of GB-speckles. The strategy of *s-LASCA* is based on the examination of an individual realization of static speckles. ^3^^,^^16^ In this case, the whole realization of the speckle field is divided into square zones; typically, each counting 5×5 or 7×7 pixels.

For each zone, the contrast of GB-speckles was calculated using the simplest formula:

$$C=\frac{\sigma_{I}}{\langle I\rangle }$$

where *I* was the varying intensity of GB-speckles, changing from point to point; *σ _I_* was the standard deviation of the intensity of fluctuations. After the contrast *C* is calculated in each point, *LASCA* image is developed. Here, the size of subarea for the local contrast calculating was 2×2 pixels. As it has been demonstrated ^14^ this size of subarea is close to optimal.

### Coloured GB speckles

To generate three two-dimensional implementations of GB speckles built for different genetic sequences, it is necessary to construct a colour image, where each colour component (red, green, and blue) has its own GB speckle structure. When all three speckle structures were totally indistinguishable, the colour images look grey-scale. If the colour components differ from each other, then, as a result, colouring will appear in the image.

**Figure 2.**
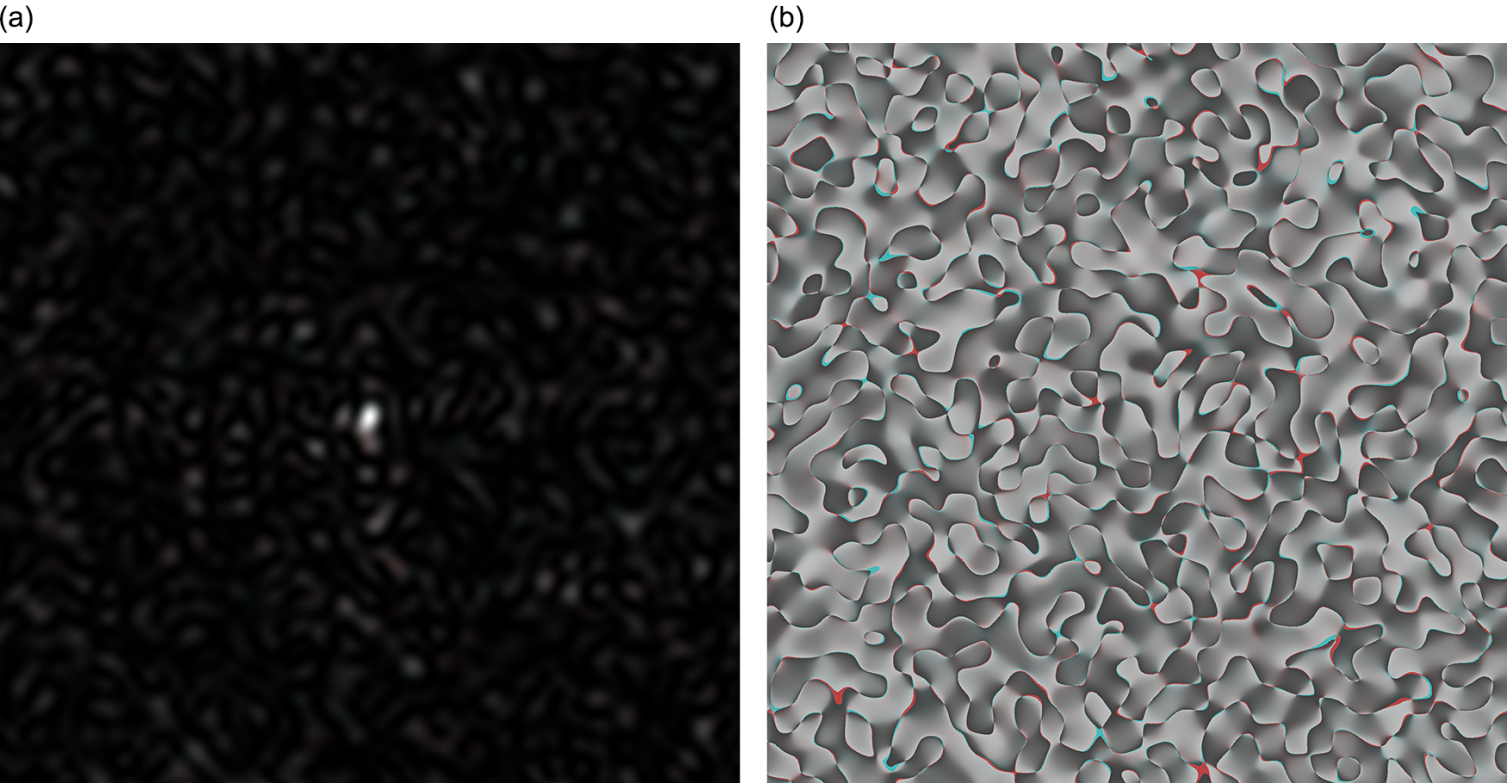
Coloured GB speckles: (a) Coloured speckle-pattern for 2D intensity distribution, (b) Coloured speckle-pattern for 2D phase distribution.

In Figure 2a, the coloured speckle-pattern for intensity distribution is presented (the red component obtained for the nucleotide sequence derived from gene #1, the green component corresponded to the nucleotide sequence of gene #2, and blue component was the relevant to gene #3 nucleotide sequence, respectively).

Figure 2a, demonstrates the differences in the initial nucleotide sequences, a slight staining appears in the colour speckle-pattern structure for a two-dimensional intensity distribution.

In Figure 2b, the coloured speckle-pattern for phase distribution is shown for such nucleotide sequences as: (i) the red component for gene #1, green for gene #2, blue for gene #3.

It is quite obvious that in the case under consideration, there is a pronounced colouring over the whole image for the field of GB-speckle.

Thus, the obtained colour image for the intensity and phase of GB speckles is a reliable diagnostic sign of the presence of polymorphism.

### A novel detection technique based on the *s-LASCA* images with coloured GB-speckles

Once an *s-LASCA* image is obtained for each of the three components of the matched genetic sequence, the final colour image can be constructed. An example of such an image is shown in Figure 3a.

**Figure 3a.**
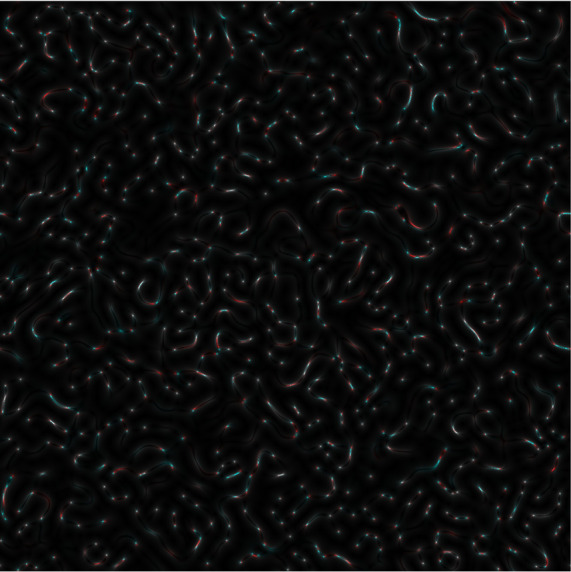
Coloured *s-LASCA* images of GB-speckles, generated for three SARS–CoV-2 genes (the gene#1, the gene#2 and the gene#3).

**Figure 3b.**
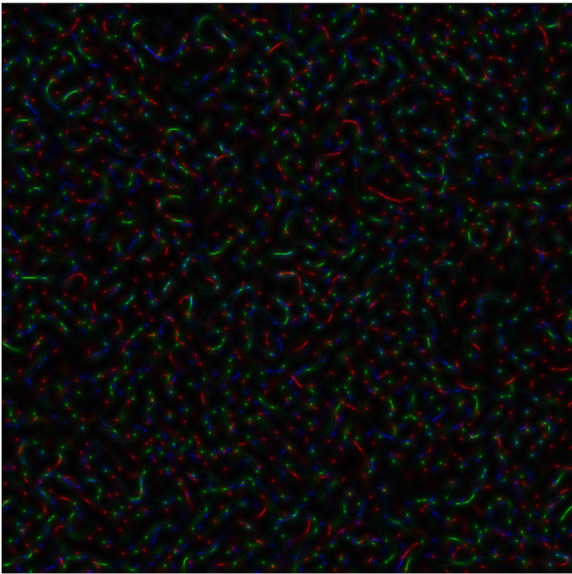
Coloured *s-LASCA* images of GB-speckles, generated for other three SARS–CoV-2 genes (the gene#4, the gene#5 and the gene#6).

## Results and discussion

It is obvious that the image shown in Figure 3a in comparison with the image in Figure 2a has a more pronounced colouring over the entire field of view, but is characterized by a higher contrast. From a quantitative point of view, the degree of colouring can be described by the value

$$R=\frac{1}{N\times M}\sum_{i=1}^{N\times M}\sqrt{\frac{1}{3}\times \frac{{\left({\mathrm{Ir}}_{i}-{\mathrm{Io}}_{i}\right)}^{2}+{\left({\mathrm{Ig}}_{i}-{\mathrm{Io}}_{i}\right)}^{2}+{\left({\mathrm{Ib}}_{i}-{\mathrm{Io}}_{i}\right)}^{2}}{{\left({\mathrm{Io}}_{i}\right)}^{2}}}$$

where ${\mathrm{Ir}}_{i}$ , ${\mathrm{Ig}}_{i}$ and ${\mathrm{Ib}}_{i}$ are values of intensity for the red, green, and blue components in each pixel,

$${\mathrm{Io}}_{i}=\frac{{\mathrm{Ir}}_{i}+{\mathrm{Ig}}_{i}+{\mathrm{Ib}}_{i}}{3}$$

is the average intensity value in each pixel, *i* is the pixel number, *M* and *N* are the number of rows and columns of the analyzed image, *N* × *M* is the total number of pixels in the image.

**Figure 3c.**
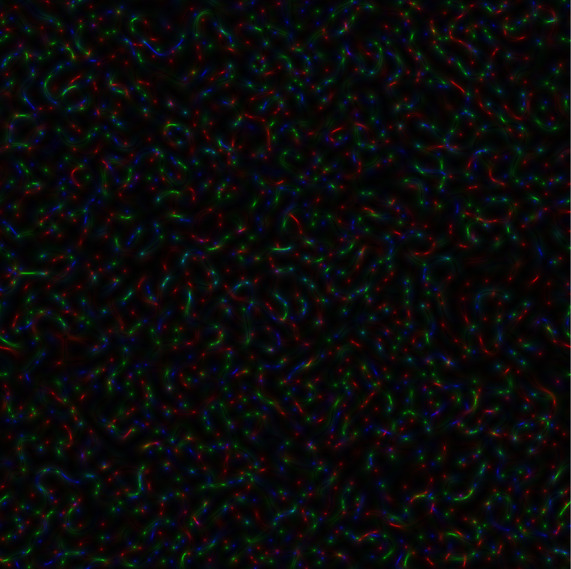
Coloured *s-LASCA* images of GB-speckles, generated for more three SARS–CoV-2 genes (the gene#5, the gene#6 and the gene#7).

Obviously, if the nucleotide sequences compared using *s-LASCA* imaging of GB-speckles are completely identical, then the three components of the resulting colour image will be identical, and therefore the value of *R* will be equal to zero. However, if there are at least minimal differences in the compared nucleotide sequences, then the value of *R* will take a positive value. Thus, the value of *R* calculated for the Figure 3a is 0.1 (gene#1, gene#2 and gene#3 are compared).

In Figure 3b, comparison of new SARS–CoV-2 genes: hCoV-19/Wuhan/WIV04/2019|2019-12-30 (gene#4), hCoV-19/England/QEUH-B11766/2020|2020-11-02 (gene#5) and hCoV19/South Africa/KRISP-EC-K005299/2020|2020-11-19 (gene#6) is presented. *R* calculated for Figure 3b equals to 0.596.

The physical meaning of the introduced parameter *R* is that this parameter characterizes the degree of coloring of the picture (GB-speckle- pattern). The bioinformatic (molecular biology) value of *R* is that it takes positive values, even in the case of the appearance of a one SNP in the analyzed nucleotide sequences. Thus, the minimum natural mutations of the virus can be determined using the parameter *R.*

Finally, three SARS–CoV-2 genes are reflected in Figure 3c (hCoV-19/England/QEUH-B11766/2020|2020-11-02 (gene#5), hCoV19/South Africa/KRISP-EC-K005299/2020|2020-11-19 (gene#6) and hCoV-19/Russia/MOS-CRIE-13604226/2020|2020-11-09 (gene#7). Again, *R* equals to 0.596 for this case.

It is important to note that the value of *R* calculated for Figure 2a and Figure 2b (coloured bare GB-speckle) equals to 0.049 and 0.026, respectively. This means that the value of *R* at least in two times higher for GB speckles, processed by *s-LASCA* imaging technique.

Evidently, *R* is positive for all images in Figures 3; so, *R* is an important diagnostic feature when detecting the presence of SNPs in SARS–CoV-2 genes. This is the main result of this paper.

## Conclusion

A fundamentally new bioinformatics technique for reliable detection of single SNPs is proposed. The new method is based on the applying of the *s-LASCA* ‘imaging technique’ generating original GB-speckles. It is established that even one SNP can be reliably detected. It has been demonstrated that suggested technique is very effective tool for discrimination between different variants of the SARS–CoV-2 spike glycoprotein gene.

## Data availability

### Underlying data

GISAID Gene: hCoV-19/cat/USA/TX-TAMU-078/2020. Accession number EPI ISL 699509;

GISAID Gene: hCoV-19/cat/Russia/RII-LEN-22246S/2021. Accession number EPI ISL 811147;

GISAID Gene: hCoV-19/cat/Greece/2K/2020. Accession number EPI ISL 717979;

GISAID Gene: hCoV-19/Wuhan/WIV04/2019. Accession number EPI ISL 402124;

GISAID Gene: hCoV-19/England/QEUH-B11766/2020. Accession number EPI ISL 642476;

GISAID Gene: hCoV19/South Africa/KRISP-EC-K005299/2020. Accession number EPI ISL 678597;

GISAID Gene: hCoV-19/Russia/MOS-CRIE-13604226/2020. Accession number EPI ISL 754198.

Sequences are available after registration at the GISAID public database.
