# Supplementary material for: Discrimination of the SARS–CoV-2 strains using of coloured s-LASCA-imaging of GB-speckles, developed for the gene “S” nucleotide sequences
Source: F1000Res. 2022 Jun 22;10:503. Originally published 2021 Jun 25. [Version 4] doi: 10.12688/f1000research.53214.4 (PMC9204187; doi:10.12688/f1000research.53214.4)
Supplement: Peer review history

# Peer review: Reviewer response for version 3

**DOI:** 10.5256/f1000research.78849.r119557

**Published:** 2022-06-16

**Authors:** Dmitry A. Zimnyakov

**Affiliations:** 1 Department of Physics, Yury Gagarin State Technical University of Saratov, Saratov, Russian Federation

I am very grateful for the chance to take part in the review of the article for your respected journal. The current manuscript is devoted to demonstrating the application of the s-LASKA method for discrimination of different variants of the SARS–CoV-2 spike gene. Fortunately, the authors consider this approach as very attractive and available for precise diagnostics of the infection with the use of a new generation of medical devices. In fact, this can be recognized as the new direction for bioscience.

 In my point of view, this is very interesting and important research that contains several new findings. The article is clearly written, conclusions correspond to data obtained, and statistical treatment is appropriate. However, the paper needs minor revision before it can be indexed. The following are my concerns about the article:

1. p. 3: ‘A new bioinformatics approach has been proposed very recently: 14 GB-speckles processing *via* an *s-LASCA* technique…’ – via should not be italic;
2. p. 7-8: the legends for Figures 1-2 contain no indication for which genes the GB-speckles were generated, as well as it is pointed in the legends for Figures 3a, 3b, and 3c. It is critical for the extended auditorium of readers to clearly understand what is demonstrated in Figures 1-2. These points need a correction.

I have no additional proposals for edits. The revised version of the article can be recommended for indexing with no further reviewing.

Is the work clearly and accurately presented and does it cite the current literature?

Yes

If applicable, is the statistical analysis and its interpretation appropriate?

Yes

Are all the source data underlying the results available to ensure full reproducibility?

Yes

Is the study design appropriate and is the work technically sound?

Yes

Are the conclusions drawn adequately supported by the results?

Yes

Are sufficient details of methods and analysis provided to allow replication by others?

Yes

Reviewer Expertise:

laser physics, biomedical science

I confirm that I have read this submission and believe that I have an appropriate level of expertise to confirm that it is of an acceptable scientific standard.

# Response

**Published:** 2022-06-17

**Authors:** Valentina Feodorova

**Affiliations:** Federal Research Center for Virology and Microbiology, Branch in Saratov, Russian Federation

Dear Prof Dmitry A. Zimnyakov,

 we highly appreciate your review for our manuscript. All your recommendations were accepted. All necessary corrections have been done in the text of the paper.

 Thank you very much once more.

 The authors.

# Peer review: Reviewer response for version 2

**DOI:** 10.5256/f1000research.76647.r93566

**Published:** 2021-09-20

**Authors:** Oleg Angelsky, Claudia Zenkova

**Affiliations:** 1 Department of Correlation Optics, Chernivtsi National University, Chernivtsi, Ukraine; 2 Chernivtsi National University, Chernivtsi, Ukraine

The paper can de accepted for indexing in the present new form.

Is the work clearly and accurately presented and does it cite the current literature?

Partly

If applicable, is the statistical analysis and its interpretation appropriate?

Yes

Are all the source data underlying the results available to ensure full reproducibility?

Yes

Is the study design appropriate and is the work technically sound?

Yes

Are the conclusions drawn adequately supported by the results?

Partly

Are sufficient details of methods and analysis provided to allow replication by others?

Yes

Reviewer Expertise:

Singular and Correlation Optics

We confirm that we have read this submission and believe that we have an appropriate level of expertise to confirm that it is of an acceptable scientific standard.

# Peer review: Reviewer response for version 2

**DOI:** 10.5256/f1000research.76647.r94278

**Published:** 2021-09-14

**Authors:** Alexey Bashkatov

**Affiliations:** 1 Department of Optics and Biophotonics, Saratov State University, Saratov, Russian Federation

The article is devoted to an extremely relevant and very  interesting topic – an application of GB- speckles, in particular, processed by the s-LASCA imaging method, in relation to the discrimination of SARS–CoV-2 strains. GB-speckle is a new word in the field of bioinformatics. In the future, these optical virtual speckle structures can be effectively used as an alternative to classical bioinformatics methods. The usage of GB-speckles can be considered as a replacement for traditional computer methods of sequencing of any nucleotide sequences.

 As it follows from the analysis of the literature, the authors of this article have successfully used these methods, based on the processing of virtual speckles, to the analysis of natural mutations in the comparing genes.

 Also, this article presents a fundamentally new method, based on the using of colored GB-speckles, processed by s-LASCA-imaging technique. The new method is based on the applying of the *s-LASCA* ‘imaging technique’ generating original GB-speckles. The article demonstrates that colored s-LASCA-imaging can be successfully used for the diagnosis and differentiation of various strains of SARS-CoV-2.

 The utilization of GB-speckles in diagnostics is an absolutely new direction, which has been developed and is being successfully promoted by the authors of this article. I have no questions about the application of speckle interferometry methods in molecular biology, reflected in this work. I only have some minor criticisms, regarding bioinformatics:

- The authors need to check carefully the reference(s) and link(s) for the bioinformatic tools used.
- After the providing of clear the answer to this question, the reviewed article can be accepted for indexing without any additional changes and without the additional cycles of reviewing.

Is the work clearly and accurately presented and does it cite the current literature?

Partly

If applicable, is the statistical analysis and its interpretation appropriate?

Yes

Are all the source data underlying the results available to ensure full reproducibility?

Yes

Is the study design appropriate and is the work technically sound?

Yes

Are the conclusions drawn adequately supported by the results?

Yes

Are sufficient details of methods and analysis provided to allow replication by others?

Yes

Reviewer Expertise:

NA

I confirm that I have read this submission and believe that I have an appropriate level of expertise to confirm that it is of an acceptable scientific standard, however I have significant reservations, as outlined above.

# Response

**Published:** 2021-09-24

**Authors:** Valentina Feodorova

**Affiliations:** Federal Research Center for Virology and Microbiology, Branch in Saratov, Russian Federation

The authors are extremely grateful to the respected reviewer Alexey Bashkatov for a detailed analysis of our article. In accordance with the recommendations of the reviewer, all references and links are checked, thank you.

# Peer review: Reviewer response for version 1

**DOI:** 10.5256/f1000research.56573.r88324

**Published:** 2021-07-16

**Authors:** Oleg Angelsky

**Affiliations:** 1 Department of Correlation Optics, Chernivtsi National University, Chernivtsi, Ukraine

The paper is devoted to the investigations of virtual Gene Based speckles for the determination the difference between SARS-Cov-2 spike glycoprotein genes. As it was noted by the authors the given method can use the classical approaches of speckle-optics. But there are some questions: 

1. Any optical speckle is the result of the interference of waves scattered by inhomogeneities, and to obtain a speckle pattern, the wavelength is decisive. Thus, the question arises about the concrete wavelengths used in the study and their relationships with pseudo inhomogeneities. It would be interesting to know about the coherence of the sources used the modelling. 
2. Please present an experiment setup, and the results of experimental modelling.
3. It would be interesting to conduct a complete analysis of the entered parameter R, to represent its physical, biochemical meaning. The virus is constantly mutating, how is it possible to estimate the degree of virus mutation using this parameter.

The paper can be indexed after reworking.

Is the work clearly and accurately presented and does it cite the current literature?

Partly

If applicable, is the statistical analysis and its interpretation appropriate?

Yes

Are all the source data underlying the results available to ensure full reproducibility?

Yes

Is the study design appropriate and is the work technically sound?

Yes

Are the conclusions drawn adequately supported by the results?

Partly

Are sufficient details of methods and analysis provided to allow replication by others?

Yes

Reviewer Expertise:

Singular and Correlation Optics

I confirm that I have read this submission and believe that I have an appropriate level of expertise to confirm that it is of an acceptable scientific standard, however I have significant reservations, as outlined above.

# Response

**Published:** 2021-07-30

**Authors:** Valentina Feodorova

**Affiliations:** Federal Research Center for Virology and Microbiology, Branch in Saratov, Russian Federation

Reply to the comments of reviewer Prof. Oleg Angelsky:

- *Any optical speckle is the result of the interference of waves scattered by inhomogeneities, and to obtain a speckle pattern, the wavelength is decisive. Thus, the question arises about the concrete wavelengths used in the study and their relationships with pseudo inhomogeneities. It would be interesting to know about the coherence of the sources used the modelling.* 
- **Response**: This is really reasonable and very important question. Fragment below is added to the new version of the text:

It is assumed that speckles are formed in the far diffraction zone and described in the Fraunhofer approximation. In this case, the expression for the amplitude of the scattered field is the Fourier transform of the field in the diffraction plane, evaluated at frequencies spaces 

 Fx=Xo/( z* λ),   Fy=Yo/( z* λ),                                (*) 

 where Xo and Yo are the coordinates in the observation plane, z is the distance between the scattering plane and the observation plane, λ is the wavelength. The illuminating radiation is completely monochromatic, thus, λ =const. 

 Reference to Goodman, J. W. Introduction to fourier optics. *McGraw Hill Companies, New York *(1988). 

 In this situation, the structure of speckles does not depend on the wavelength and z. Only the sizes of GB-speckles depend on these values, the average size of which is determined by the ratio: 

 d~3*z* λ /a                                            (**) 

 where a is the size of the illuminated fragment of virtual surface. It is important to emphasize that the ratio λ/a characterizes the diffraction angular divergence of a laser beam in the far field, and the product of this divergence angle by the light traveled distance z is equal to the lateral size of the beam. 

 New reference: M. Francon. La granularite laser (speckle) et ses applications en optique. Masson, Paris, New York, Barcelone, Milan 1978. 

 Thus, it can be seen that the diameter of the undisturbed laser beam (namely, this value is on the right side of the expression (**)) and the average speckle size are approximately equal to each other in any observation plane. 

 In other words, when the parameters z and λ change, a proportional change in the size of all speckles occurs synchronously. At the same time, the structure of speckle-patterns in all observation planes are completely similar, only their scale changes from plane to plane, but not the shape of the speckles or their location in the speckle pattern. 

  

- *Please present an experiment setup, and the results of experimental modelling. *
- **Response: **Experimental studies were not carried out in this work, only computer modeling. The scheme for calculating GB speckles during radiation diffraction on a virtual scattering surface is described in detail in the work:  Ulianova OV, et al.: Speckle-interferometry and speckle-correlometry of GB-speckles. Frontiers in Bioscience-Landmark 2019; 24, 700-711. This fact is mentioned in the new version of this article. 
- *It would be interesting to conduct a complete analysis of the entered parameter R, to represent its physical, biochemical meaning. The virus is constantly mutating, how is it possible to estimate the degree of virus mutation using this parameter.  *
- **Response: **The physical meaning of the introduced parameter R is that this parameter characterizes the degree of coloring of the picture (GB-speckle- pattern). The bioinformatic (molecular biology) value of R is that it takes positive values, even in the case of the appearance of a one SNP in the analyzed nucleotide sequences. Thus, the minimum natural mutations of the virus can be determined using the parameter R. This circumstance is also noted in the Conclusions of new version of this article. 

​​​​​The authors are extremely grateful to the respected reviewer, whose critical comments have significantly improved the quality of this article.
